# Inhibitor of apoptosis proteins are potential targets for treatment of granulosa cell tumors – implications from studies in KGN

**DOI:** 10.1186/s13048-019-0549-6

**Published:** 2019-08-14

**Authors:** Konstantin Bagnjuk, Verena Jasmin Kast, Astrid Tiefenbacher, Melanie Kaseder, Toshihiko Yanase, Alexander Burges, Lars Kunz, Doris Mayr, Artur Mayerhofer

**Affiliations:** 1 Biomedical Center Munich (BMC), Cell Biology, Anatomy III, Ludwig-Maximilians-University (LMU), Grosshaderner Strasse 9, 82152 Planegg, Germany; 2 Department of Endocrinology and Diabetes Mellitus, Faculty of Medicine, Fukuoka University, 7-45-1 Nanakuma, Jonan-ku, Fukuoka City, Japan; 3 Frauenklinik Campus Großhadern, Ludwig-Maximilians-University (LMU), Marchioninistraße 15, 81377 Munich, Germany; 4 Division of Neurobiology, Department Biology II, Ludwig-Maximilians-University (LMU), Grosshaderner Strasse 9, 82152 Planegg, Germany; 5 Institute for Pathology, Ludwig-Maximilians-University (LMU), 80337 Munich, Germany

**Keywords:** Cell death, Ovarian granulosa cell tumor, Apoptosis

## Abstract

**Background:**

Granulosa cell tumors (GCTs) are derived from proliferating granulosa cells of the ovarian follicle. They are known for their late recurrence and most patients with an aggressive form die from their disease. There are no treatment options for this slowly proliferating tumor besides surgery and chemotherapy. In a number of tumors, analogs of the second mitochondria-derived activator of caspases (SMAC), alone or in combination with other molecules, such as TNFα, are evolving as new treatment options. SMAC mimetics block inhibitor of apoptosis proteins (IAPs), which bind caspases (e.g. XIAP), or activate the pro-survival NF-κB pathway (e.g. cIAP1/2). Expression of IAPs by GCTs is yet not fully elucidated but recently XIAP and its inhibition by SMAC mimetics in a combination therapy was described to induce apoptosis in a GCT cell line, KGN. We evaluated the expression of cIAP1 in GCTs and elucidated the effects of the SMAC mimetic BV-6 using KGN as a model.

**Results:**

Employing immunohistochemistry, we observed cIAP1 expression in a tissue microarray (TMA) of 42 GCT samples. RT-PCR confirmed expression of cIAP1/2, as well as XIAP, in primary, patient-derived GCTs and in KGN. We therefore tested the ability of the bivalent SMAC mimetic BV-6, which is known to inhibit cIAP1/2 and XIAP, to induce cell death in KGN. A dose response study indicated an EC_50_ ≈ 8 μM for both, early (< 8) and advanced (> 80) passages, which differ in growth rate and presumably aggressiveness. Quantitative RT-PCR showed upregulation of NF-κB regulated genes in BV-6 stimulated cells. Blocking experiments with the pan-caspase inhibitor Z-VAD-FMK indicated caspase-dependence. A concentration of 20 μM Z-VAD-FMK was sufficient to significantly reduce apoptosis. This cell death was further substantiated by results of Western Blot studies. Cleaved caspase 3 and cleaved PARP became evident in the BV-6 treated group.

**Conclusions:**

Taken together, the results show that BV-6 is able to induce apoptosis in KGN cells. This approach may therefore offer a promising therapeutic avenue to treat GCTs.

**Electronic supplementary material:**

The online version of this article (10.1186/s13048-019-0549-6) contains supplementary material, which is available to authorized users.

## Background

Apoptosis can be activated via two different pathways, the extrinsic death receptor pathway and the intrinsic mitochondria associated pathway [1, 2]. Both will execute apoptosis by cleaving and therefore activating caspases, which in turn degrade other proteins, based on their peptidase activity [3]. The intrinsic pathway is induced by the release of pro-apoptotic molecules from mitochondria, for example cytochrome c, endonuclease G, apoptosis inducing factor (AIF), high temperature requirement protein A2 (HtrA2 also known as OMI) or the second mitochondria-derived activator of caspases (SMAC; or its murine homolog, known as direct inhibitor of apoptosis protein binding protein with low PI (DIABLO)) [2, 4].

SMAC blocks inhibitor of apoptosis proteins (IAPs, e.g. baculoviral IAP repeat containing protein 1–8, BIRC1–8), which are highly expressed in various tumors [5]. Therefore, IAPs are potential targets in oncology. Different SMAC mimetics have been developed to target IAPs [6–8]. Some of these compounds are monovalent and others are bivalent. The latter target two BIR domains simultaneously and have been shown to be more potent [6, 9].

*BIRC2* (cIAP1), *BIRC3* (cIAP2), and *BIRC4* (XIAP) are expressed in granulosa cells of ovarian follicles [10]. Tumors, which arise from these cells (granulosa cell tumors (GCTs)), are often steroidogenic and produce estrogen in prepubertal (juvenile GCTs) and postmenopausal woman (adult GCTs) [11]. Adult GCTs usually bear the FOXL2(C134W) mutation. Although these tumors are steroidogenic, it remains unknown whether they grow in a gonadotropin-dependent manner, as shown for other tumors [12, 13].

The majority of patients who suffer from aggressive or recurrent GCTs, where the aggressiveness and probability of relapse is not reflected histologically, die from their disease [11]. Due to the low proliferation speed, chemotherapy is often ineffective and therefore surgery is the only promising way to treat GCTs. In other ovarian malignancies, such as epithelial cancers, chemotherapy is more effective. In these tumors it was shown that reoccurrence might be due to reduced immune-surveillance or drug-resistant cells [14, 15]. In GCTs this option was never discussed but might be of interest in the rare case of effective first line chemotherapy.

To improve the situation for GCT-patients, it is important to develop alternative methods. A widely used model to study this type of tumor is the KGN cell line [16]. These cells are steroidogenic and bear the FOXL2 mutation. It was recently shown that *BIRC2* (cIAP1) and *BIRC4* (XIAP) are expressed in GCTs and in KGN [17]. The examined samples were, however, only very weakly stained for *BIRC2* (cIAP1) and reportedly negative for *BIRC3* (cIAP2). Immunostaining results, as those mentioned, may depend on fixation, antibody specificity and antibody concentration. Negative results do not necessarily rule out expression. Furthermore, in granulosa cells of healthy woman *BIRC2*, *BIRC3* and *BIRC4* expression levels vary during follicular development [18]. Of note, GCTs are thought to stem from proliferating GCs of unknown follicular stage. In the present study we therefore examined expression of BIRC2 (cIAP1) in 42 GCTs. We next evaluated the actions of the bivalent SMAC mimetic BV-6, which targets XIAP and cIAP1/2 simultaneously, in studies employing KGN.

## Results

### cIAP1 protein expression in GCTs

**Fig. 1:**
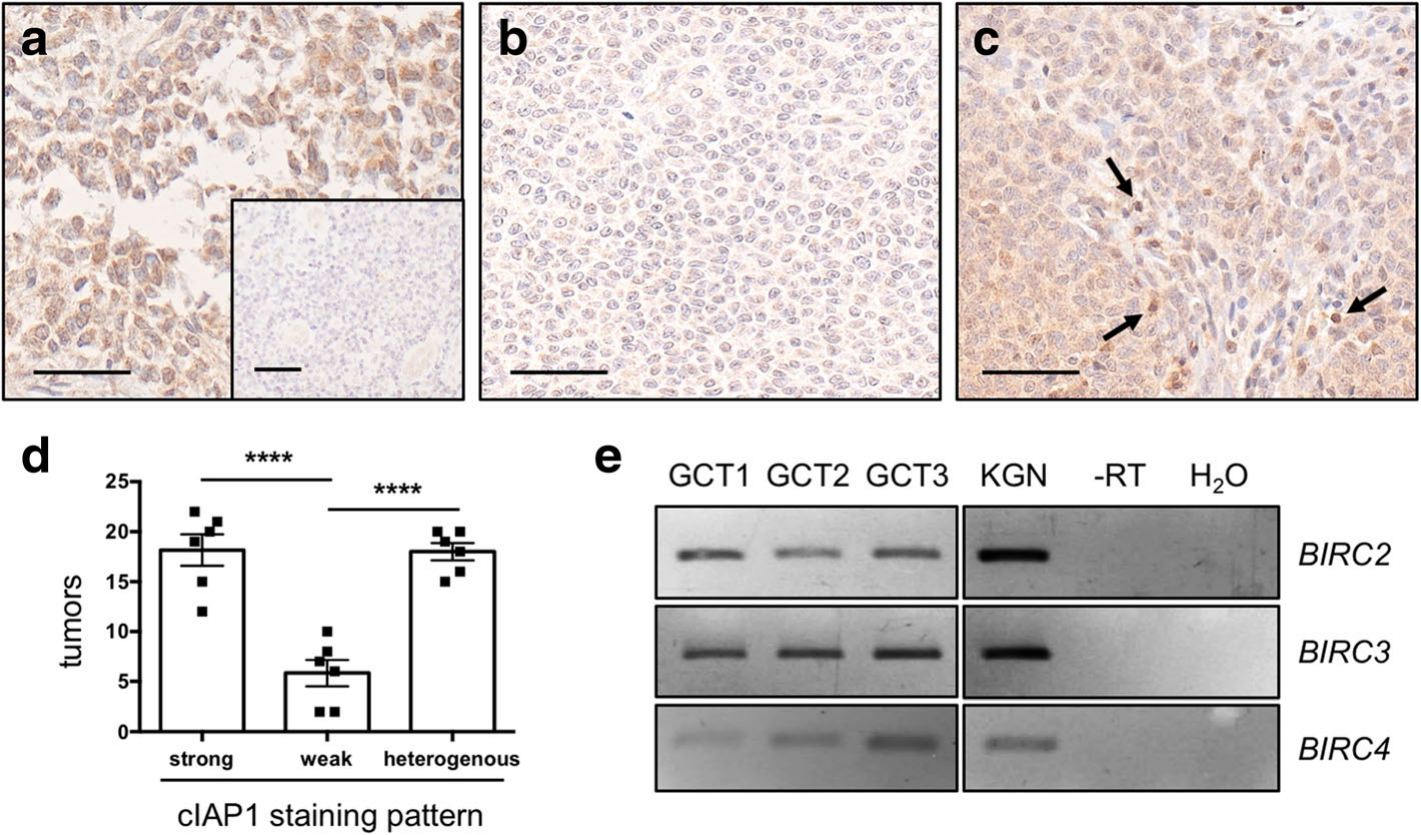
*BIRC2* (cIAP1)*, BIRC3* and *BIRC4* expression in GCTs and KGN. Identification of *BIRC2*, *BIRC3* and *BIRC4* expression at mRNA level in KGN and GCTs and evaluation of cIAP1 protein expression in GCTs. (**a**-**d**) Expression of cIAP1 in 42 different samples of GCTs were analyzed by immunohistochemistry. (**a**,**d**) Results of the evaluation, performed by 6 researchers, indicated strong and homogenous cIAP1 staining in 18.2 tumors (mean). (**b**,**d**) 5.8 tumors (mean) showed weak cIAP1 staining. (**c**,**d**) Heterogeneous cIAP1 staining was visible in 18.0 tumors (mean). Arrows indicate strongly stained nuclei. (**a**-**c**) Negative control (rabbit serum) showed no staining (**a**, inset). Scale bars correspond to 50 μM. (**d**) Shown are the mean values and SEM. (**e**) RT-PCR of three independent GCTs and KGN (primers are described in Table 1). Controls consisted of a no reverse transcription (−RT) sample and a no template (H_2_O) sample

We examined cIAP1 expression in 42 ovarian GCTs using a specific anti-cIAP1 antibody. In general, three distinct staining patterns were observed: strong, weak and heterogeneous (Fig. 1a-d). Classification of the tumor-staining by 6 individuals revealed 18.2 +/− 1.6 (43.3%) samples with homogenously strong cIAP1 expression (Fig. 1a,d), whereas weak expression was observed in only 5.8 +/− 1.3 tumors (13.9%) (Fig. 1b,d). Heterogeneous staining was found in 18.0 +/− 0.9 samples (42.9%) (Fig. 1c,d). In these samples, some cells showed strong nuclear cIAP1 staining (indicated by arrows), some remained unstained, and others showed weak staining in the cytoplasm. A significant difference (****p < 0.0001) was found between the number of strongly versus weakly stained tumors and between the number of heterogeneously versus weakly stained tumors. Negative controls (non-immune rabbit serum instead of antiserum) showed only marginal and non-specific staining (Fig. 1a, Inset).

### *BIRC2, BIRC3* and *BIRC4* mRNA is expressed in GCTs and KGN

To elucidate gene expression of IAPs in primary GCT-samples, we conducted a RT-PCR analysis using the listed primers (Table 1). *BIRC2*, *BIRC3*, and *BIRC4* gene expression levels were analyzed in three human GCTs, as well as in KGN. All targets were found in the tested samples (Fig. 1e). The band intensities varied across different tumors, indicating patient-specific expression levels.

**Table 1:** List of oligonucleotide primers used for RT-PCR studies

Target	Sequence (5′ – 3′)		Reference	Product size (bp)
*BIRC2*	Forward	GAC ATC ATC ATT GCG ACC CAC	NM_001166.4	192
	Reverse	TGG TTT CCA AGG TGT GAG TAC T		
*BIRC3*	Forward	AGA ACA CCT GAG ACA TTT TCC CA	NM_001165.4	202
	Reverse	GAC ATC ATC ATC GTT ACC CAC A		
*BIRC4*	Forward	TGT GGA GGA GGG CTA ACT GA	NM_001167.3	83
	Reverse	AGA TAT TTG CAC CCT GGA TAC CA		
*TNFα*	Forward	ATG AGC ACT GAA AGC ATG ATC C	NM_000594.4	217
	Reverse	GAG GGC TGA TTA GAG AGA GGT C		
*MCP-1*	Forward	AGG TGA CTG GGG CAT TGA T	NM_002982.3	109
	Reverse	GCC TCC AGC ATG AAA GTC TC		
*IL8*	Forward	TCT TGG CAG CCT TCC TGA	NM_000584.4	271
	Reverse	GAA TTC TCA GCC CTC TTC		
*L19*	Forward	AGG CAC ATG GGC ATA GGT AA	NM_000981.3	199
	Reverse	CCA TGA GAA TCC GCT TGT TT		
*HPRT*	Forward	CCT GGC GTC GTG ATT AGT GA	NM_000194.2	163
	Reverse	GGC CTC CCA TCT CCT TCA TC		
*PPIA*	Forward	AGA CAA GGT CCC AAA GAC	NM_021130.5	118
	Reverse	ACC ACC CTG ACA CAT AAA		
*TBP*	Forward	TGC ACA GGA GCC AAG AGT GAA	NM_003194.5	132
	Reverse	CAC ATC ACA GCT CCC CAC CA		

### BV-6 induces time-dependent cell death in KGN

**Fig. 2:**
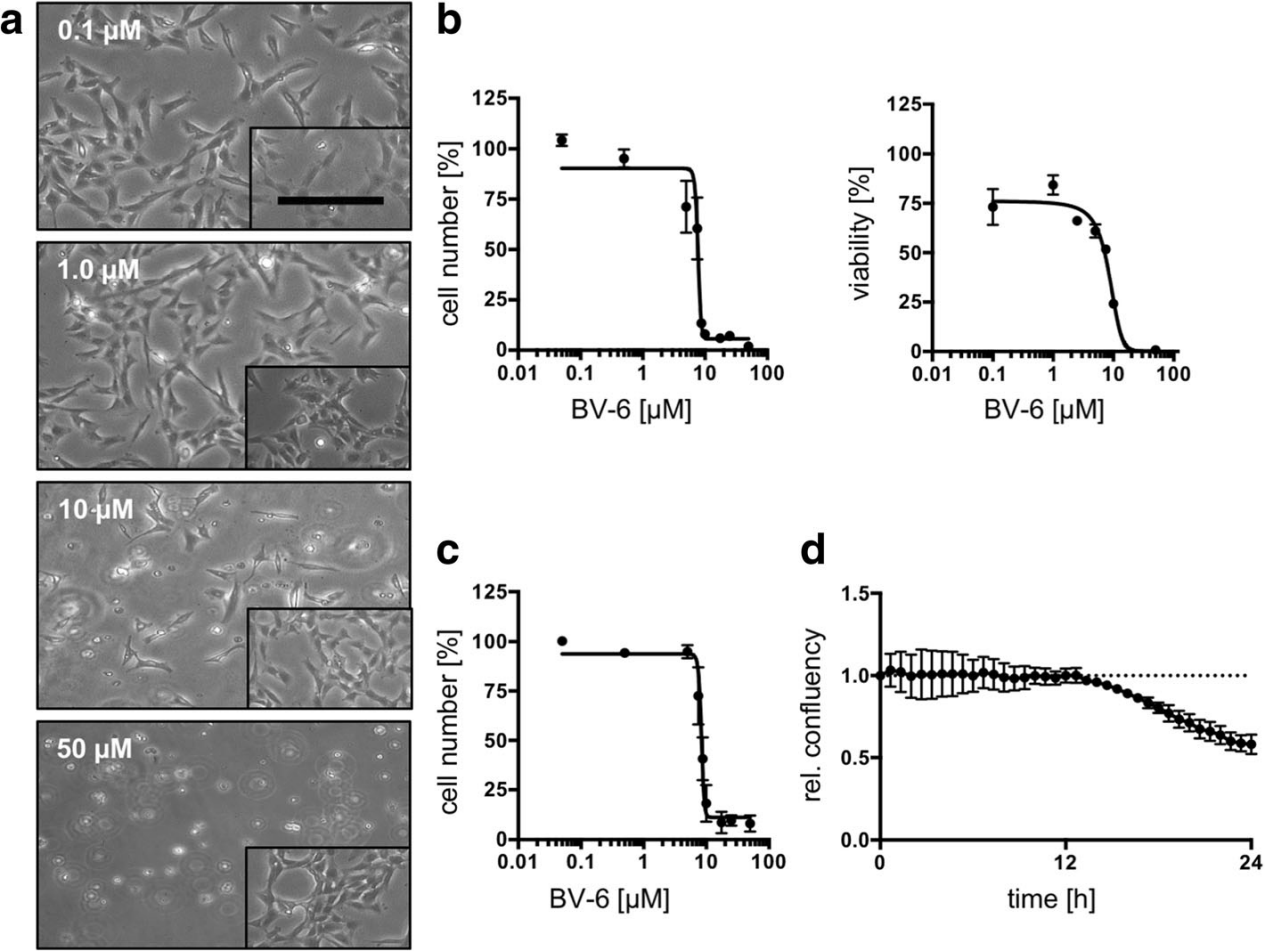
Effects of BV-6 treatment on KGN. The SMAC mimetic BV-6 was used in different cell viability assays to explore the effects on KGN. Different BV-6 concentrations (0.05–100 μM) were tested. (**a**) Live cell images of KGN (passages > 80) treated with BV-6 (0.1, 1, 10 and 50 μM) for 24 h. Corresponding solvent controls are shown in insets. At 0.1 and 1 μM no indications for cell death were evident. At 10 μM detached cells were visible. At 50 μM all cells detached. Scale bar indicates 50 μm. (**b**, left graph) Subsequent cell counting analysis of KGN (passages > 80) treated with BV-6 (0.05–100 μM) revealed an EC_50_ of 7.4–8.2 μM after nonlinear regression analysis. (*n* = 3, bars indicate SEM). (**b**, right graph) ATP assay with BV-6 (0.1–100 μM) confirmed the cell counting experiment. Nonlinear regression analysis revealed an EC_50_ ranging from 7.2 to 9.7 μM. (*n* = 4, error bars indicate SEM). (**c**) To examine a possible involvement of the passage number, KGN from early passages (< 8) were stimulated with BV-6 (0.05–100 μM) for 24 h and then counted. Nonlinear regression analysis revealed an EC_50_ of 8.1 - 8.6 μM. (**d**) time dependence was evaluated by confluency measurement (20 min intervals) of BV-6 (EC_50_, 8 μM) treated KGN. Data were normalized to the solvent control. Decline in confluency started after 12 h and reached 0.58 (control =1.0) after 24 h. (*n* = 3, error bars indicate SEM, rel. = relative)

IAPs are potential targets for tumor treatment. Therefore, we tested the ability of BV-6, a bivalent SMAC mimetic, to induce cell death in KGN [6]. This cell line is known to divide faster in high passages, implicating elevated aggressiveness [19]. Therefore, we examined BV-6 actions in KGN from high (> 80, Fig. 2) and low passages (< 8). (A flowchart of the experiments can be found in the Additional file 1: Figure S1a). Using live cell imaging, we tested 4 concentrations of BV-6, ranging from 0.1 to 50 μM (Fig. 2a), in comparison to the respective solvent controls (Fig. 2a insets). At low concentrations of BV-6 (0.1 μM, 1 μM) cells were not affected within the timeframe of 24 h. At 10 μM, BV-6 induced cell death, as seen by detached and floating cells. This effect was intensified when 50 μM of BV-6 was used. The concentration-dependent effects were independent of the passage number (data not shown).

To determine the half maximal effective concentration (EC_50_) after 24 h, KGN (passages > 80) were treated with increasing concentrations of BV-6 for 24 h and then counted (Fig. 2b). As expected, at low concentrations (0.1–5.0 μM) cells remained unaffected. However, concentrations above 5 μM reduced cell numbers. Nonlinear regression analysis described a sigmoidal curve and interpolation revealed an EC_50_ for BV-6 ranging from 7.4 μM to 8.2 μM (Fig. 2b, left graph). For the low passages, we found an EC_50_ ranging between 8.1 and 8.6 μM (Fig. 2c). As the effective concentrations did not substantially vary between high and low passages, all further experiments were implemented on KGN at high passages (> 80).

To further test the defined EC_50_, cell viability was analyzed using an ATP assay (Fig. 2b, right graph). For this purpose, cells were treated with different concentrations of BV-6 (0.1 to 50 μM) and evaluated after 24 h. In accordance with the results in Fig. 2b, nonlinear regression analysis revealed a half-maximal reduction of viability by 7.2 to 9.7 μM BV-6. Live cell imaging and subsequent measurement of confluency were assessed in BV-6 (EC_50_, 8 μM) treated KGN. The space occupied by cells (confluency) relative to the maximally available space, was measured in 20 min intervals. Confluency began to decline after 12 h and reached 0.58 after 24 h, as compared to the control (1.0). This result is in line with the other experiments. Taken together, we found an EC_50_ (24 h) of approximately 8 μM for BV-6 in KGN, which is passage-independent but time-dependent.

### BV-6 leads to NF-κB activation and apoptosis in KGN

BV-6 is known to block XIAP, cIAP1 and cIAP2 and therefore is able to activate apoptosis through different pathways, including NF-κB activation and direct caspase activation [6]. To examine these possibilities in KGN, we exposed KGN to BV-6 (EC_50_) for 24 h and examined involvement of NF-κB pathway and caspase activation.

**Fig. 3:**
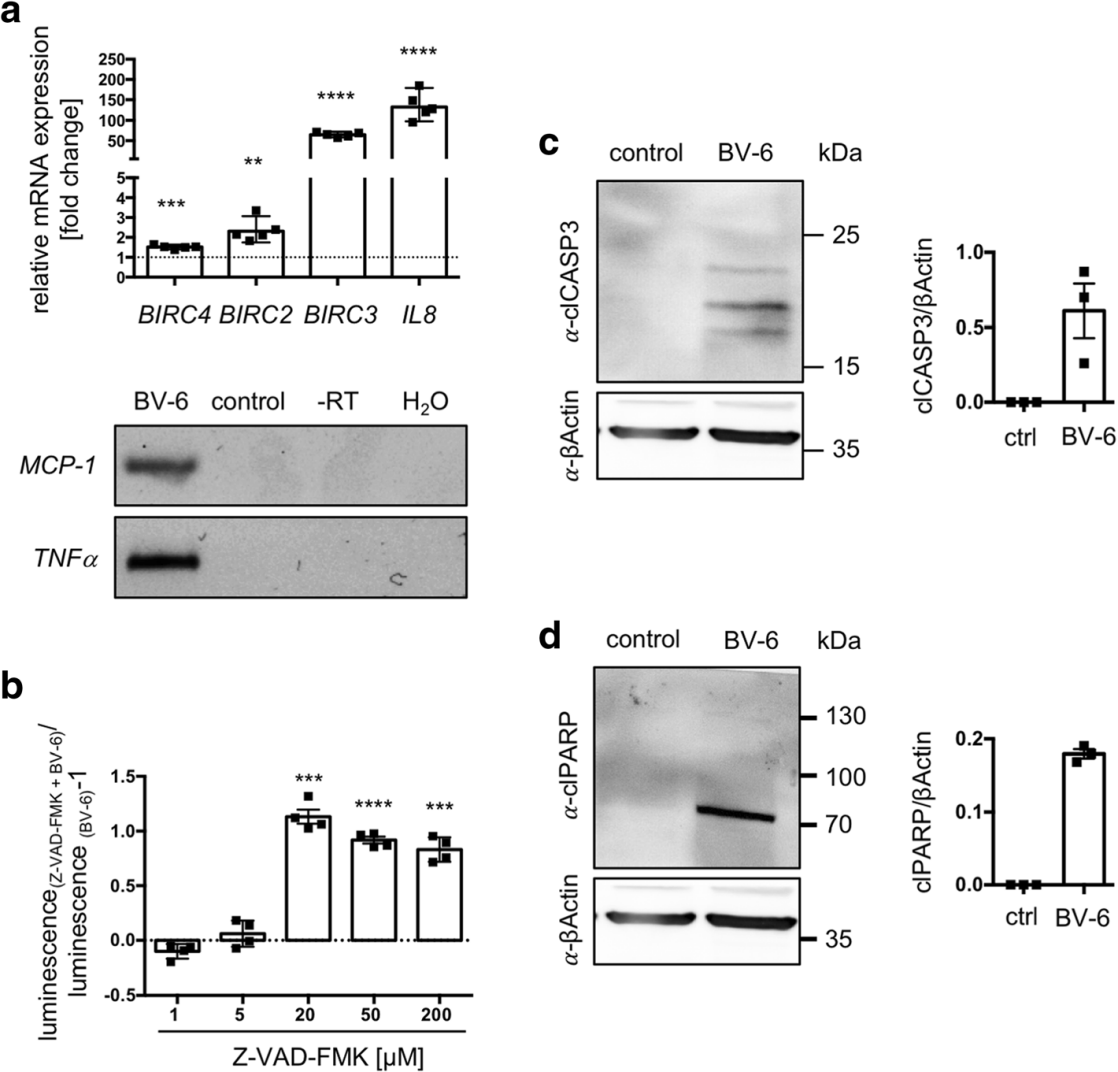
Classification of cell death induced by BV-6 in KGN. Results of qRT-PCR, ATP-assay and Western Blot experiments performed to classify BV-6 induced cell death. (**a**) To test NF-κB pathway activation, qRT-PCR experiments of known target genes (*BIRC4*, *BIRC2*, *BIRC3, IL8*, *TNFα* and *MCP-1*) were performed. (**a**, upper graph) *BIRC4*, *BIRC2*, *BIRC3* and *IL8* levels were significantly increased after BV-6 stimulation (*n* = 5, geometric mean with 95% confidence interval, **p < 0.01, ***p < 0.001, ****p < 0.0001). (**a**, lower panels) *MCP-1* and *TNFα*, both were absent in untreated samples but expressed in BV-6-treated samples (*n* = 5). Agarose gel shows a representative picture. The controls lacked template (H_2_O) or reverse transcriptase (−RT control). (**b**) ATP-assay was conducted in KGN that were stimulated with a combination of Z-VAD-FMK (1, 5, 20, 50, 200 μM) and BV-6 (EC_50_, 8 μM) for 24 h. Afterwards luminescence was normalized to cells solely treated with BV-6 (EC_50_, 8 μM). 1 μM and 5 μM Z-VAD-FMK had no effect on BV-6 induced cell death. 20 μM, 50 μM and 200 μM Z-VAD-FMK significantly reduced BV-6 induced cell death. (n = 4, mean and SEM, ***p < 0.001, ****p < 0.0001). (**c**,**d**) Western Blot analysis using specific α-clCASP3-, α-clPARP- and α-βActin -antibodies. KGN, treated with BV-6 (EC_50_, 8 μM) for 24 h were analyzed in comparison to controls. Western Blot revealed cleavage of (**c**) CASP3 and (**d**) PARP. The solvent treated controls lacked any apoptosis relevant signal but the loading control showed equal loading (*n* = 3, error bars indicate SEM)

*IL8*, *MCP-1*, *TNFα*, *BIRC2*, *BIRC3* and *BIRC4* are known NF-κB target genes [20–23]. Therefore, upregulation points to pathway activation. Indeed, all of the tested genes were expressed in BV-6 stimulated KGN (Fig. 3a). *BIRC4*, *BIRC2*, *BIRC3* and *IL8* were upregulated with a fold-change rate of 1.52 +/− 0.04, 2.4 +/− 0.26, 64.9 +/− 2.57 and 133.5 +/− 14.9, respectively (**p < 0.01 for *BIRC2,* ***p < 0.001 for *BIRC4* and ****p < 0.0001 for *BIRC3* and *IL8*). *TNFα* and *MCP-1* were not found in untreated KGN but were present in BV-6 treated samples, as shown in the representative agarose gel (Fig. 3a).

To test involvement of caspases, different concentrations of Z-VAD-FMK in combination with BV-6 (EC_50_, Fig. 3b) were added to KGN cultures. Z-VAD-FMK is a pan-caspase inhibitor and therefore functions as a blocker of apoptosis [24]. Cell viability was assessed using ATP assays. At low concentrations (1 μM and 5 μM) Z-VAD-FMK had no effect on BV-6 induced cell death. Interestingly, viability was significantly improved at higher doses (20 μM ***p < 0.001, 50 μM ****p < 0.0001 and 200 μM ***p < 0.001).

Next we screened BV-6-treated KGN for apoptotic markers by Western Blot. We used a specific anti-cleaved caspase 3 (α-clCASP3) antibody to detect the intermediate step of apoptosis and anti-cleaved poly (ADP-ribose)-polymerase (α-clPARP) antibody to detect the terminal step of apoptosis [1, 25]. Both, clCASP3 (Fig. 3c) and clPARP (Fig. 3d) were present in three independent samples of BV-6 (8 μM)-treated KGN but completely absent from the corresponding controls. Using an anti-βActin antibody, equal loading was confirmed.

## Discussion

A hallmark of cancer is resistance to cell death [26], which may be related to altered IAP expression profiles. cIAP1 and cIAP2, for example, have been shown to be overexpressed in various tumors, including cervical cancer, esophageal squamous cell carcinoma, hepatocarcinoma, medulloblastoma, several forms of lung cancer, pancreatic cancer and many more, as summarized by Dubrez et al. [27]. SMAC mimetics, which target intracellular IAPs, are for this reason a hot topic in cancer biology. Some of these compounds are currently being tested in phase I and phase II clinical trials and show promising results [28]. To our knowledge (https://www.clinicaltrials.gov), there are several ongoing clinical studies with SMAC mimetics in different solid tumors and lymphomas, however not in GCTs.

Which IAPs are expressed by GCTs is not fully known. Yet a recent work described strong and homogenous expression of XIAP but a weak cIAP1 expression in GCTs [17]. Immunostaining results may depend on fixation, antibody specificity, as well as many other factors. Therefore, we tested a validated and specific anti-cIAP1 antibody in 42 tumor samples and employed primary GCT samples and KGN for RT-PCR studies. While expression of cIAP1 was strong in most tested GCT samples, the second largest group showed heterogeneous staining. Only a small fraction (13.9%) was weakly stained.

Furthermore, we detected *BIRC3* (cIAP2) mRNA, which in the previous study was not described [17], but was shown to be upregulated in many other tumors [27]. However, in line with the previous study by Leung et al., *BIRC4* (XIAP) was detected. Thus, GCTs are endowed with several IAPs, which render them targets for SMAC mimetics.

Given that *BIRC2, 3* and *4* are present, we hypothesized that BV-6 may be of special interest [6]. BV-6 targets cIAP1/2 and XIAP, which block apoptosis by directly inhibiting caspases (e.g. XIAP = BIRC4), and by activating the pro-survival canonical NF-κB pathway (e.g. cIAP1/2 = BIRC2/3) [6]. We tested BV-6 actions in KGN, a cell line that is widely used for the study of GCTs [16]. NF-κB was shown to play a role in BV-6 mediated apoptosis [6]. BV-6 stimulation increased the levels of several target genes of this pathway, namely *IL8*, *MCP-1*, *TNFα*, *BIRC2*, *BIRC3* and *BIRC4* in KGN [20–23]. Interestingly, the increase in expression of the 3 tested IAPs varied, with *BIRC3* (cIAP2) showing the strongest effect (fold change = 133.5). It may be possible that upregulation of *BIRC2* and *BIRC3* are cellular attempts to counter apoptosis [21]. TNFα plays an important role in BV-6 induced cell death in many tumor cell lines and is expressed upon BV-6 stimulation in KGN. Therefore, TNF receptor mediated activation of NF-κB and apoptosis are likely to be involved in BV-6 actions in KGN [6]. As we did not elucidate the whole pathway, e.g. RIP1 ubiquitination and cIAP1/2 degradation, we can not conclude to the full mode of action of BV-6. However, it is likely that cIAP1/2, which ubiquitinate RIP1, are degraded. In turn this may lead to deubiquitylation of RIP1 and subsequently activation of apoptosis or necroptosis through a TNFα autocrine loop [6, 29, 30].

Passaging KGN induces a change in proliferation speed, which might be related to tumor aggressiveness [19]. We tested low and advanced passages for their sensitivity to BV-6 but found no substantial differences. BV-6 -induced cell death in KGN was concentration- (EC_50_ ≈ 8 μM) and time-dependent. Low concentrations of BV-6 (e.g. 1 μM) induced cell death in KGN, but longer incubation times (72 h) were needed (Additional file 1: Figure S1b).

In a recent study, a SMAC mimetic alone was reported incapable to induce cell death in KGN [17], but co-applied with a PPARγ agonist it became effective. The PPARγ pathway was shown to be relevant in cancer earlier [31, 32]. In the previous study the bivalent SMAC mimetic “compound A”, originally developed by the pioneering group around Vince [7] was employed. This compound was designed to bind the same IAP domains (BIR2-BIR3) as BV-6, but is chemically different, which could explain the disparate potency [6, 7]. Our results obtained with BV-6 are in line with published work, verifying that some SMAC mimetics induce cell death as single agents in various cancers [6, 33–37].

The Western Blot experiments of our study showed cleaved caspase 3, indicating an intermediate step of apoptosis and cleaved PARP, i.e. a terminal step of apoptosis in BV-6-treated KGN. Furthermore, blocking experiments with the pan-caspase blocker Z-VAD-FMK improved KGN viability [24]. Hence, we conclude that BV-6-induced cell death is mainly apoptosis. Necroptosis (regulated necrosis) has also been described in literature to occur upon SMAC mimetic stimulation in some settings [38]. It is postulated that different cell death pathways are interlinked and might be triggered simultaneously [39]. We attempted to determine a possible involvement of necroptosis and examined phosphorylated MLKL (T357/S358) in BV-6-treated samples by Western Blot, using a validated phosphospecific antibody [40]. Using this marker, we were not able to find signs for necroptotic cell death in KGN. MLKL was evident in every sample but phosphorylation and subsequently oligomerization were not observed (Additional file 1: Figure S1c).

The results obtained in cellular studies are in line with reports showing that apoptosis is induced by BV-6 in many cell types [6], but the effects of different SMAC mimetics in distinct experiment settings and cells may vary. For example, it was found that a SMAC mimetic monotherapy is effective only in a small number of tumor cell lines (< 15%), whereas in combination with exogenously added TNFα or TRAIL, around 50% of the tumor cell lines died [41]. Further, the dependence on cytokines was proposed. In mouse models, for example, it was shown that next to the innate immune system the adaptive immune system plays an important role in SMAC mimetic-mediated cell death [42]. The fact that these compounds not only affect the tumor cell itself but also trigger effector cells like natural killer cells, was shown recently [43]. Lecis and group further supported this hypothesis, as SMAC mimetics affected the tumor niche by exerting immunomodulatory effects on macrophages [44]. Thus, next to their effect on tumor cells, SMAC mimetics have a broad impact on the tumor microenvironment and the immune system. These points can however not be examined in cellular experiments with KGN cells.

In the present work we focused on the SMAC mimetic BV-6 as a potential inducer of apoptosis in KGN. As described, apoptosis is either induced extrinsically (death receptor pathway) or intrinsically (mitochondrial pathway). The extrinsic pathway is executed upon binding of a death ligand (e.g. TNFα) to the corresponding receptor (e.g. TNFR1). Then initiator caspase (caspase 8) is activated, which leads to further cascaded reactions including caspase 3 activation and cleavage of crucial proteins, including PARP [25, 45]. These steps were readily observed in BV-6-treated KGN and indicate that BV-6 does induce apoptosis.

Taken together, our results may imply that GCTs, which as we found are often positive for cIAP1, albeit in a patient-depended manner, can possibly be treated by BV-6. The results further suggest that prior to treatment, testing for expression of IAPs may be useful and should be performed.

## Conclusions

The SMAC mimetic BV-6 is able to induce apoptosis in KGN, which express XIAP and cIAP1/2. The results imply that these IAPs, if present in primary tumors, may serve as targets for therapeutic approaches.

## Methods

### Human GCT samples

The ethical committee of the LMU has approved the study (project 390–15) and patients had agreed to the use of the tissue. Samples (approximately 0.5 cm^3^) from three patients (age 41, 53 and 66 years) undergoing surgery were obtained. Tumor cells were enriched by collagenase digestion in the presence of antibiotics (1% penicillin/streptomycin (Biochrom GmbH, Berlin, Germany)) and subsequent plating on culture wells (HAM’s-F12 supplemented with 10% fetal calf serum (FCS)), as described for primary human granulosa cell cultures [26]. RNA was extracted and subjected to RT-PCR.

All patients were diagnosed at the Institute for Pathology (LMU, Munich). The diagnoses were confirmed by an experienced gynecologic pathologist (D.M.).

In addition, two tissue microarrays (TMA) were assembled and used. Archival material of 42 patients with GCT was available. All patients were treated surgically at the same institution (Department of Gynecology, University of Munich). Tissue biopsies (*n* = 42) were taken from representative regions of paraffin-embedded tumor samples (donor) and arrayed into a new recipient paraffin block by using MTA-1 (Micro Tissue Arrayer) from Beecher Instruments, USA.

### Culture and treatment of KGN

The human ovarian granulosa-like tumor cell line KGN was obtained from the Riken BioResource Research Centre (Ibaraki, Japan) [16] and the use of this patented cell line was approved by T. Yanase. KGN were cultured in Dulbecco’s Modified Eagle Medium/Nutrient Mixture F-12 (DMEM/F-12, Thermo Fisher Scientific, Waltham, MA, USA) supplemented with 10% FCS (Capricorn Scientific GmbH, Ebsdorfergrund, Germany) and 1% penicillin/streptomycin (Biochrom GmbH, Berlin, Germany) in T75 flasks (Thermo Fisher Scientific, Waltham, MA, USA) under constant temperature (37 °C) and CO_2_ concentration (5%). As KGN change in aggressiveness over culture time [19] we used low passages (< 8) and high passages (> 80) for preliminary experiments. As a passage dependence was absent in terms of sensitivity to BV-6 we proceeded with high passages (> 80).

To rule out serum effects, experiments were executed in serum-free DMEM/F-12 media. Therefore, KGN were stimulated for 24 h either with the second mitochondria-derived activator of caspases (SMAC) mimetic BV-6 (0.05–100 μM; Selleck Chemicals LLC, Houston, TX, USA) alone or in combination with the pan-caspase inhibitor Z-VAD-FMK (1–200 μM; Selleck Chemicals LLC, Houston, TX, USA). BV-6 and Z-VAD-FMK were dissolved in water and DMSO (Merck, Darmstadt, Germany), respectively. Solvent controls were included in every experiment. To block caspases before cell death induction, Z-VAD-FMK was applied 2 h prior to BV-6 administration. All experiments were carried out at least 3 times, if not described otherwise.

### RT-PCR and qRT-PCR

RT-PCR was conducted as previously described [46, 47]. In brief, total RNA was extracted using RNeasy Plus Micro Kit (Qiagen, Hilden, Germany) and employed for reverse transcription. RT-PCR and qRT-PCR primers (Table 1) were designed using Primer3 and synthesized by metabion international AG (Planegg, Germany) [48]. The qRT-PCR analyses were performed utilizing the QuantiFast SYBR Green PCR Kit (Qiagen, Hilden, Germany). Samples (final cDNA concentration 10 ng/reaction) were measured in duplicates in a LightCycler® 96 System (Roche Diagnostics, Penzberg, Germany; melting at 95 °C for 5 min, followed by 40 cycles of denaturation at 95 °C for 10 s and extension at 60 °C for 30 s, and a final melting step with continuous heating (0.5 °C/s from 65 °C to 97 °C) and a cool-down step at 37 °C for 30 s). Results were calculated using the 2^−ΔΔCq^ method and expression was normalized to ribosomal protein L19 *(L19)*, hypoxanthine Phosphoribosyltransferase 1 (*HPRT)*, peptidylprolyl isomerase A (*PPIA)* and TATA-box binding protein (*TBP)* as endogenous references. After PCR, products were separated and visualized on a 2% agarose gel. Expected bands were cut out, sequenced (GATC Biotech AG, Konstanz, Germany) and analyzed using BLAST tool [49].

### Immunohistochemistry of ex vivo GCT using anti-cIAP1 antibody

Immunohistochemical staining of cIAP1 proteins to examine expression patterns in GCTs was done as described in previous work [46, 47]. Two slides of the TMAs, containing a total of 42 tumors in duplicates, were used for this study. After deparaffinizing, the heat-induced epitope retrieval (HIER) method was applied to retrieve antigens. Afterwards endogenous peroxidase was blocked with a methanol (10%)/H_2_O_2_ (3%) solution. Unspecific binding was reduced by 10% goat serum in PBS. To detect cIAP1 protein, a polyclonal antibody was used (HPA005513, Sigma Aldrich, St. Louis, MO, USA). As a negative control rabbit serum replaced the primary antiserum. As a secondary antibody a biotinylated goat anti-rabbit antibody (111–065-144, Jackson Immuno Research, Cambridge, UK) was used. After complexing avidin with biotin (ABC reaction) and staining by means of 3,3′ diaminobenzidine tetrahydrochloride (DAB), the slides were counterstained with hematoxylin. Images were captured using a Zeiss Axiovert microscope with an Insight Camera (18.2 Color Mosaik). The TMAs were evaluated by 6 different researchers, who classified each tumor into one of the three groups (homogenously strong staining, weak staining/no staining or heterogeneous staining).

### Confluency measurement and live cell imaging

Confluency was measured as described before [40, 47]. In brief, 3 × 10^5^ KGN plated on a 60 mm^2^ culture dish (Sarstedt AG & Co. KG, Nümbrecht, Germany) were stimulated with BV-6 (EC_50_, 8 μM) or the solvent control and monitored for 24 h using the JuLi™ Br Live Cell Analyzer (NanoEnTek Inc., Seoul, Korea). Pictures were captured every 20 min and confluency was evaluated using the in-built algorithm. The experiments were repeated 3 times.

Images of KGN, stimulated with 0.1, 1, 10, 50 μM BV-6, were also captured using a cell culture microscope with an 10X objective (Leica Biosystems, Wetzlar, Germany).

### Viability measurements using cell counting and ATP-assay

EC_50_ was identified using 2 different methods. First a cell counting experiment was carried out with low passages (< 8) and high passages of KGN (> 80). Therefore 1.5 × 10^5^ cells were seeded on 6 well plates (Sarstedt AG & Co. KG, Nümbrecht, Germany) and incubated over night at 37 °C and 5% CO_2_. On the next day the cells were stimulated with BV-6 concentrations ranging from 0.05 to 100 μM or the corresponding solvent control for 24 h. Afterwards cells were trypsinized and counted using the CASY® counting system (OMNI Life Science GmbH & Co KG, Bremen, Germany). Cell counts were normalized to solvent controls. Experiments were repeated at least 3 times.

The CellTiter-Glo® ATP assay Kit (Promega, Mannheim, Germany) was perfomed following the manufacturer’s instructions. In brief 1 × 10^4^ cells/well (KGN passages > 80) were seeded in 96-well plates (Sarstedt AG & Co. KG, Nümbrecht, Germany) and incubated at 37 °C and 5% CO_2_. The next day culture media was replaced by colorless media without supplements. After 2 h, BV-6 (0.1–50 μM) was added and cells were incubated for a time period of 24 h. Further, BV-6 (8 μM) treatment was accompanied by different Z-VAD-FMK concentrations ranging from 0 to 200 μM. Z-VAD-FMK was applied 2 h before BV-6 stimulation to block caspases. Luminescence was measured using a plate reader (FLUOstar Optima, BMG Labtech, Ortenberg, Germany).

### Verification of apoptosis hallmarks by Western blot

Apoptosis is a form of cell death that is executed by caspases. Cleavage and therefore activation of caspase 3 is known to be an intermediate step, which leads to cleavage of proteins including PARP that is known to be a terminal step of apoptosis. Therefore, we treated KGN (10^6^ cells/60 mm^2^ dish (Sarstedt AG & Co. KG, Nümbrecht, Germany)) with BV-6 (EC_50_, 8 μM) for 24 h. Afterwards we generated crude protein extracts using RIPA buffer. Western Blot was carried out as described before [40, 46]. 20 μg/lane protein were loaded on 12% SDS-PAGE gels. To detect cleaved caspase 3 a specific α-clCASP3 antibody (#9664, Cell Signaling Technology, Denvers, MA, USA) was used. To detect a cleavage product of PARP the specific α-clPARP antibody (#56255, Cell Signaling Technology, Denvers, MA, USA) was applied. As a loading control an α-βActin antibody (A5441, Sigma Aldrich, St. Louis, MO, USA) was used. For decoration of bound primary antibody horseradish peroxidase (HRP) conjugated goat α-rabbit or goat α-mouse antibody (Jackson Immuno Research, Cambridge, UK) were used.

### Statistics and graphs

Cell culture and live cell imaging pictures were evaluated using FIJI [50]. Construction and statistical evaluation of graphs, including nonlinear regression analyses, were done in Prism 6 (GraphPad, San Diego, CA, USA). The statistical significance between different staining patterns of tumors was evaluated by one-way ANOVA (Tukey; Geisser-Greenhouse correction). Effectiveness of Z-VAD-FMK on BV-6-induced cell death and mRNA expression of NF-κB regulated genes were evaluated statistically by one sample *t*-tests. Final figures were constructed using PowerPoint for Mac 2013 (Microsoft, Redmond, WA, USA).

## Additional file

Additional file 1: **Figure S1.** Workflow of experiments and effects of BV-6 on KGN. (a) Schematic workflow of experiments: First EC_50_ after 24 h was determined by cell counting and ATP assay in KGN (passage > 80) and by cell counting in a low passage of KGN (< 8). All further experiments were carried out with the determined EC_50_ and with KGN of higher passages (> 80). Afterwards a Z-VAD-FMK dilution experiment was carried out, using KGN that were treated with BV-6(EC_50_,). (b) Live cell imaging experiment of stimulated KGN (BV-6, 1 μM) versus the corresponding control for 72 h. The low concentration caused a time-dependent effect by reducing number of attached cells. Scale bar = 100 μm (c) Western Blot of BV-6 (EC_50_, 8 μM)-stimulated KGN and the corresponding control. An antibody against phosphorylated (p) MLKL(T357/S358) (ab187091, Abcam, Cambridge, UK) and one against MLKL (ab184718, Abcam, Cambridge, UK) were examined to explore possible induction of necroptosis. MLKL bands were visible, whereas the necroptosis marker (pMLKL) was absent. (TIFF 8189 kb)

## Data Availability

Upon request.

## Abbreviations

AIF: Apoptosis inducing factor
ATP: Adenosine triphosphate
BIR: Baculoviral IAP repeats
BIRC: Baculoviral IAP repeat containing protein
CASP8: Caspase 8
cIAP: Cellular inhibitor of apoptosis protein
DAB: 3,3′ diaminobenzidine tetrahydrochloride
DIABLO: Direct inhibitor of apoptosis protein binding protein with low PI
EC_50_: Half maximal effective concentration
FCS: Fetal calf serum
FOXL2: Forkhead Box L2
GCTs: Granulosa cell tumors
HIER: Heat-induced epitope retrieval
HPRT: Hypoxanthine phosphoribosyltransferase 1
HRP: Horseradish peroxidase
HRP: Horseradish peroxidase
HtrA2: High temperature requirement protein A2
IAPs: Inhibitor of apoptosis proteins
IL8: Interleukin 8
KGN: Human ovarian granulosa-like tumor cell line
L19: ribosomal protein L19
MCP-1: Monocyte chemoattractant protein-1
MLKL: Mixed lineage kinase domain-like protein
mRNA: Messenger ribonucleic acid
PPIA: Peptidylprolyl isomerase A
TBP: TATA-box binding protein
α-clCASP3: anti-cleaved caspase 3
α-clPARP: anti-cleaved poly (ADP-ribose)-polymerase
