# Supplementary figures and images for: Inhibitor of apoptosis proteins are potential targets for treatment of granulosa cell tumors – implications from studies in KGN
Source: J Ovarian Res. 2019 Aug 14;12:76. doi: 10.1186/s13048-019-0549-6 (PMC6694575; doi:10.1186/s13048-019-0549-6)

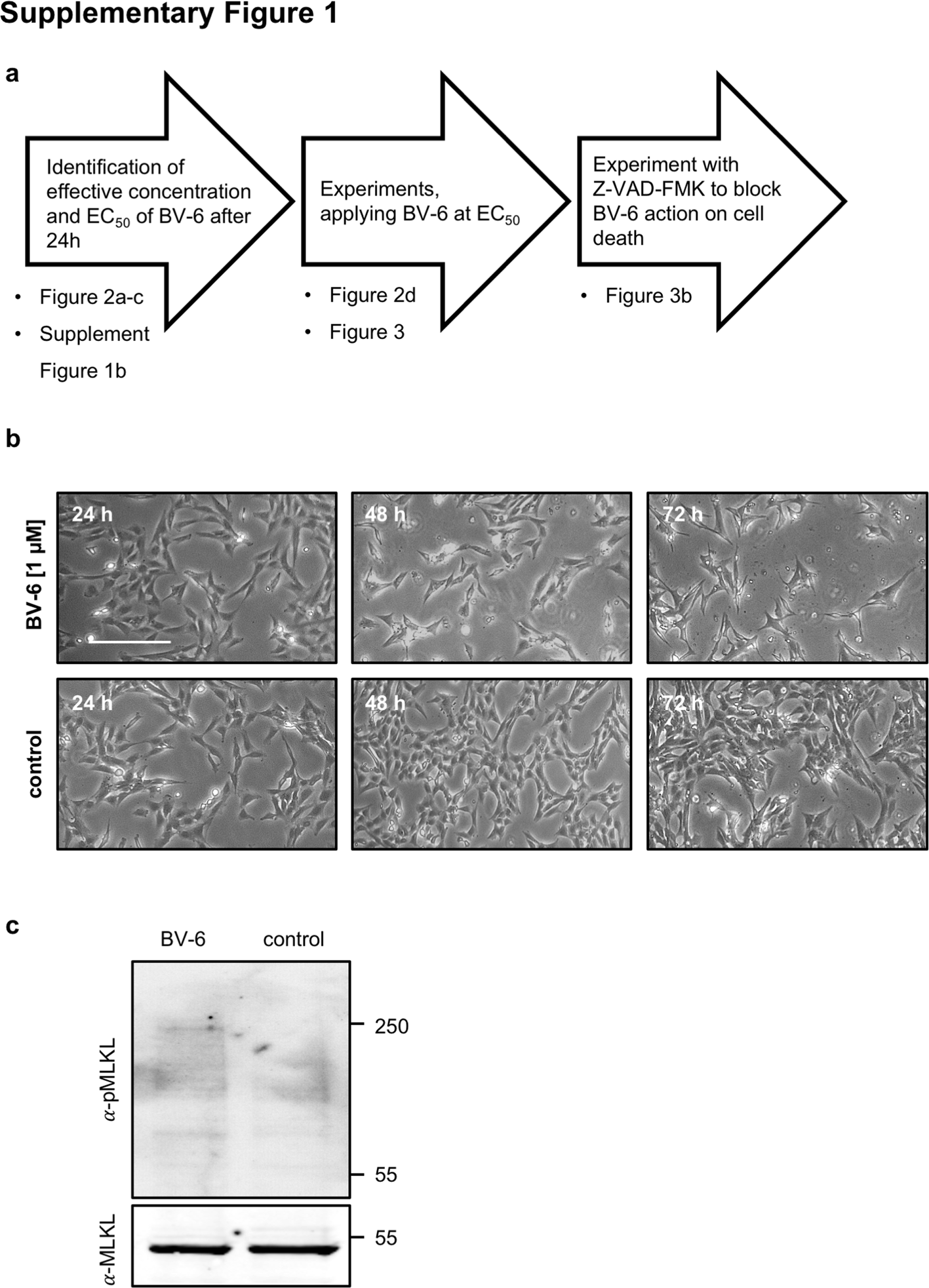

Supplement: Supplementary file 1 — Figure S1. Workflow of experiments and effects of BV-6 on KGN. (a) Schematic workflow of experiments: First EC50 after 24 h was determined by cell counting and ATP assay in KGN (passage > 80) and by cell counting in a low passage of KGN (< 8). All further experiments were carried out with the determined EC50 and with KGN of higher passages (> 80). Afterwards a Z-VAD-FMK dilution experiment was carried out, using KGN that were treated with BV-6(EC50,). (b) Live cell imaging experiment of stimulated KGN (BV-6, 1 μM) versus the corresponding control for 72 h. The low concentration caused a time-dependent effect by reducing number of attached cells. Scale bar = 100 μm (c) Western Blot of BV-6 (EC50, 8 μM)-stimulated KGN and the corresponding control. An antibody against phosphorylated (p) MLKL(T357/S358) (ab187091, Abcam, Cambridge, UK) and one against MLKL (ab184718, Abcam, Cambridge, UK) were examined to explore possible induction of necroptosis. MLKL bands were visible, whereas the necroptosis marker (pMLKL) was absent. (TIFF 8189 kb) [file 13048_2019_549_MOESM1_ESM.tiff]
